# Colorimetric Detection of Some Highly Hydrophobic Flavonoids Using Polydiacetylene Liposomes Containing Pentacosa-10,12-diynoyl Succinoglycan Monomers

**DOI:** 10.1371/journal.pone.0143454

**Published:** 2015-11-23

**Authors:** Deokgyu Yun, Daham Jeong, Eunae Cho, Seunho Jung

**Affiliations:** 1 Department of Bioscience and Biotechnology, Microbial Carbohydrate Resource Bank (MCRB) & Center for Biotechnology Research in UBITA (CBRU), Konkuk University, Seoul, South Korea; 2 Institute for Ubiquitous Information Technology and Applications (UBITA) & Center for Biotechnology Research in UBITA (CBRU), Konkuk University, Seoul, South Korea; National Cancer Institute at Frederick, UNITED STATES

## Abstract

Flavonoids are a group of plant secondary metabolites including polyphenolic molecules, and they are well known for antioxidant, anti-allergic, anti-inflammatory and anti-viral propertied. In general, flavonoids are detected with various non-colorimetric detection methods such as column liquid chromatography, thin-layer chromatography, and electrochemical analysis. For the first time, we developed a straightforward colorimetric detection system allowing recognition of some highly hydrophobic flavonoids such as alpha-naphthoflavone and beta-naphthoflavone, visually using 10,12-pentacosadiynoic acid (PCDA) derivatized with succinoglycan monomers isolated from *Sinorhizobium meliloti*. Besides changes in visible spectrum, we also demonstrate fluorescence changes using our detection system in the presence of those flavonoids. The succinoglycan monomers attached to PCDA molecules may function as an unstructured molecular capturer for some highly hydrophobic flavonoids by hydrophobic interactions, and transmit their molecular interactions as a color change throughout the PCDA liposome.

## Introduction

Flavonoids are polyphenolic compounds synthesized in plants as secondary metabolites, and exist in fruits, drinks, vegetables, and biological fluids. Since they have a numbers of therapeutic and pharmacological properties such as being antioxidants, antibacterial, or anti-inflammatory, they have drawn the interest of many researchers [1]. Although various analytical strategies for flavonoid detection are available using column liquid chromatography (LC), thin-layer chromatography (TLC), and electrochemical analysis [2], the colorimetric detection system have not been reported.

There are a number of flavonoids worth mentioning as they were studied in our system: as a model flavonoid, naringenin, is recognized for its antioxidant properties and has been studied for reducing cholesterol level [3]. In addition, the flavonoid has been reported to form a complex with beta-cyclodextrin (βCD) which is a cyclic alpha-1,4 linked glucan composed of 7 glucosidic unit and well-known host molecule for hydrophobic molecules [4]. A highly hydrophobic flavonoid, alpha-naphthoflavone (ANF) is known as a chemopreventive agent and a potent aromatase inhibitor [5]. Its structural isomer, beta-naphthoflavone (BNF) is also considered as a chemopreventive agent, and known as an inducer of cytochromes P450 detoxification enzymes [6].

Succinoglycans produced by *Sinorhizobium meliloti* have a the repeating unit which is composed of the octasaccharide subunits [Glcβ-1,3-Glcβ-1,3-Glcβ-1,6-Glcβ-1,6-Glcβ-1,4-Glcβ-1,4-Glcβ-1,3-Galβ(α)-1] with several substituents such as pyruvyl, acetyl, and succinyl groups [7]. Succinoglycan monomers (SGM) can be classified further as monomer 1 (SGM1), monomer 2 (SGM2), and monomer 3 (SGM3), according to the numbers of succinate moiety on each molecule. Although succinoglycan monomers are linear octasaccharides unlike βCD, they can effectively complex with scarcely soluble drugs such as salicylic acid and pindolol [8, 9]. In addition, succinoglycans have been investigated for the enantiomeric separation of some flavonoids as chiral additives [10, 11]. It was through this known molecular interaction between succinoglycan monomers and flavonoids that we were inspired to perform a targeted molecular design.

As we were interested in developing novel methods in flavonoid detection, we considered 10,12-pentacosadiynoic acid (PCDA), a reagent with known stimuli-responsive properties. PCDA can undergo photopolymerization via an 1,4-addition reaction on UV irradiation at 254 nm. The resultant polydiacetylens (PDAs) have a conjugated polymer structure with alternating ene-yne sequence and display characteristic electronic and optical properties due to the presence of the highly delocalized π-electron system in the polymer backbone. External stimuli such as pH, heat, solvent, and molecular recognition can induce conformational changes in PDAs accompanied with blue to red color transition [12–14]. Furthermore, the red-phase PDAs show fluorescence around 550 and 640 nm of wavelength, while the initially polymerized blue-phase PDAs lack the property [15]. Using these features, label-free PDA based chemo/biosensor systems have been developed through functionalizing an antibody, probe DNA, and enzymes [16–19]. Recent study had been reported that extracellular polysaccharides (EPS) interact with specific flavonoids, signal molecules in promoting the nodulation by Rhizobiaceae family [10, 20, 21]. Accordingly, we investigated a PCDA-mediated colorimetric detection for flavonoids using succinoglycan monomers, the major components of EPS produced by *S*. *meliloti*.

## Materials and Methods

### Purification of succinoglycan monomers isolated from *S*. *meliloti* Rm 1021

*S*. *meliloti* Rm 1021 was grown in GMS medium at 30°C for 5 days [7]. *S*. *meliloti* was supplied from the Microbial Carbohydrate Resource Bank (MCRB) at Konkuk University, Korea. After culturing the cells, they were removed by centrifugation and the supernatant was concentrated five-fold by rotary evaporation. After adding three volumes of ice-cold ethanol, extracellular polysaccharides (EPS) were precipitated. After removing the EPS, to purify succinoglycan monomers, the supernatant was again concentrated, this time five-fold from the previous concentrated volume. Succinoglycans were then precipitated by adding seven volumes of ethanol. The precipitate was dissolved in distilled water. After centrifugation, the supernatant was concentrated and a Bio-gel P6 column (2.5 cm × 145 cm) was used for purification. The monomers, dimers, and trimers of the succinoglycan subunit were separated with size exclusion chromatography (SEC). The monomers were further fractionated as monomer 1 (SGM1), monomer 2 (SGM2), and monomer 3 (SGM3) fractions using a DEAE Sephadex A-25 column. The monomers were eluted with KCl in 10 mM MOPS buffer using a linear gradient from 5 mM to 250 mM KCl. The individual monomers SGM1, SGM2, and SGM3 were collected and desalted using a Bio-gel P4 column.

### Synthesis of pentacosa-10,12-diynoyl succinoglycan monomers

#### *N*-Hydroxysuccinimide ester of 10,12-pentacosadiynoic acid (NHS-PCDA)

1g of PCDA (2.7 mmol) was dissolved in 10 ml of dichloromethane. *N*-hydroxysuccinimide (NHS) (337.5 mg, 2.9 mmol)), and 1-Ethyl-3-(3-dimethylaminopropyl)carbodiimide (EDC) (615 mg, 3.2 mmol) were added to the mixture solution [19]. The resulting organic solution was stirred for 4 h at ambient temperature. After evaporation of the solvent in vacuo, the product was extracted using ethyl acetate and water. To remove remaining water, magnesium sulfate was added to the organic layer. The organic solvent part was separated by centrifugation and filtering, and then the solvent was removed in vacuo. The product was powdered and obtained in 612 mg. The product was confirmed using thin-layer chromatography (TLC, hexane/ethyl acetate 3:1).

#### Synthesis of *N*-(2-aminoethyl)-10,12-pentacosadiynamide using NHS-PCDA and ethylenediamine (NH_2_-PCDA)

NHS-PCDA (380 mg, 0.8 mmol) was dissolved in 4 ml of dichloromethane. To the organic solvent, 1 ml of ethylenediamine were added [22]. The mixture solution was stirred at ambient temperature for 8 h. After stirring, the product solution was extracted with water and dichloromethane in ratio 2:1. Then the organic layer was dried with magnesium sulfate and filtered, and the solvent was removed by evaporation. The product was then crystallized and the white product was obtained in 204 mg. The product was confirmed with electrospray ionization mass spectrometry (ESI-MS) and thin-layer chromatography (TLC, hexane/ethyl acetate 3:1).

#### Reductive amination with succinoglycan monomers and *N*-(2-aminoethyl)-10,12-pentacosadiynamide

NH_2_-PCDA 26 mg (60 μmol) was dissolved in dimethyl sulfoxide (DMSO) 700 μl at 65°C for 15 min [23]. To the solution, acetic acid 300 μl and sodium cyanoborohydride 31.5 mg (500 μmol) was added with stirring at 65°C for 10 min. The succinoglycan monomers, SGM1 14.5 mg (10 μmol), SGM2 15.5 mg (10 μmol), and SGM3 16.5 mg (10 μmol) was added to the solution. The reaction mixture was stirred at 65°C for 4 h. The resulting product was precipitated with acetone. The precipitate was dissolved in water and dialyzed with dialysis tubing (MWCO 1000). The final lyophilized products, pentacosa-10,12-diynoyl succinoglycan monomers (SGM1-PCDA, SGM2-PCDA, and SGM3-PCDA), were analyzed by MALDI-TOF and NMR spectroscopy.

### Matrix-assisted desorption/ionization time-of-flight mass spectrometry (MALDI-TOF MS)

The mass spectrum was obtained using a MALDI-TOF mass spectrometer (Voyager-DE^™^ STR BioSpectrometry, PerSeptive Biosystems, Framingham MA, USA) in the negative-ion mode. 2, 4, 6-trihydroxyacetophenone (THAP) was used as the matrix.

### Nuclear magnetic resonance (NMR) spectroscopy

For the NMR spectroscopic analysis, we used a Bruker Avance 500MHz spectrometer (AMX, Germany) to record the ^1^H NMR spectra. NMR analyses were performed in D_2_O at room temperature.

### Preparation of PDA liposomes containing pentacosa-10,12-diynoyl succinoglycan monomers or mono[6-deoxy-6-(pentacosa-10,12-diynyl amidomethyl)]-β-cyclodextrin

PCDA (5.04 mg, 0.9 mM) was dissolved in 1 ml of chloroform, and the organic solvent was removed by flowing with N_2_ [24]. Then, thin lipid film was obtained on the glass surface. Pentacosa-10,12-diynoyl succinoglycan monomers (SGM-PCDA) or mono[6-deoxy-6-(pentacosa-10,12-diynyl amidomethyl)]-β-cyclodextrin (βCD-PCDA) with HEPES buffer solution (5 mM, pH 8.0) were added to give a total lipid concentration of 1 mM. Synthesis of βCD-PCDA was carried out as described previously [25, 26]. The samples were heated at 80°C for 20 min, and sonicated for 15 min using probe sonicator (Sonics VC-505, USA) at 40% power. The warm solution was filtered through a 0.8 μm filter (Sartorius Stedim Biotech, Minisart, Germany) to remove undispersed lipid, and the liposome milky solution was cooled to 4°C. Polymerization was achieved at room temperature by irradiating the solution with UV lamp at 254 nm for 10 min. Polymerization of milky solutions from each SGM-PCDA and PCDA (1:9) resulted in blue-phase PDA molecules (SGM1-PDA, SGM2-PDA, and SGM3-PDA). The solution of PDA liposomes containing βCD-PCDA was also polymerized by UV irradiation (βCD-PDA).

### Dynamic light scattering (DLS)

DLS measurements were carried out with a DynaPro Plate Reader (Wyatt Technology Corporation, CA, USA) at constant room temperature.

### UV-Vis spectroscopy

A Shimadzu Corporation UV 2450, UV-Vis spectrophotometry from 400 to 900nm was used to evaluate the blue to red color transition of the PDAs and SGM-PDA in response to presence of beta-naphthoflavone at room temperature.

### Fluorescence spectroscopy

Since the blue to red color change of the PDAs occurs with generation of fluorescence, fluorescence intensity was observed by a fluorescence microplate reader (Spectramax Gemini EM, Molecular Devices) at room temperature. Fluorescence spectra were measured after addition of analyte for 40 min. The excitation wavelength was 485 nm, excitation and emission band widths were 9 nm.

### Colorimetric response (CR %) measurement

PDA vesicles have blue to red color change when response occurs; and the degree of color change could be characterized by colorimetric response (CR%), which calculated with following equation [27]: CR% = [(PB_0_– PB_1_)/PB_0_] x 100%, where PB = A_640 nm_ /(A_640 nm_ + A_550 nm_); A_640 nm_ is the absorbance at 640 nm (blue wavelength); A_550 nm_ is the absorbance at 550 nm (red wavelength); PB_0_ and PB_1_ are values calculated before and after color change, respectively. The experiments were done with at least three replicates.

### Fluorescence change on the concentration of β-naphthoflavone and kinetic study of CR%

Colorimetric detection test on the concentration of β-naphthoflavone and kinetic study of CR% were achieved as follows: To confirm colorimetric sensitivity of SGM-PDA for flavonoids, fluorescence study was performed according to β-naphthoflavone concentration (at 0.09375, 0.1875, 0.375, 0.75, 1.5 and 3 mM). Then, a kinetic study for the CR% change was done for 2 h (at 2mM β-naphthoflavone). As control group, PDA only liposome solution was used. SGM1-PDA, SGM2-PDA, and SGM3-PDA were used as experimental groups.

### Partition coefficient determination

The partition coefficient determination (log *P*) value is defined as a logarithm of the ratio of the concentrations of a chemical in the octanol relative to its concentration in the water [28]. The log *P* values of flavonoids, known as a parameter value about lipophilicity, were investigated using ACD/Percepta Platform (ACD/labs, Toronto, Canada).

## Results

### Isolation and purification of succinoglycan monomers

**Fig 1:**
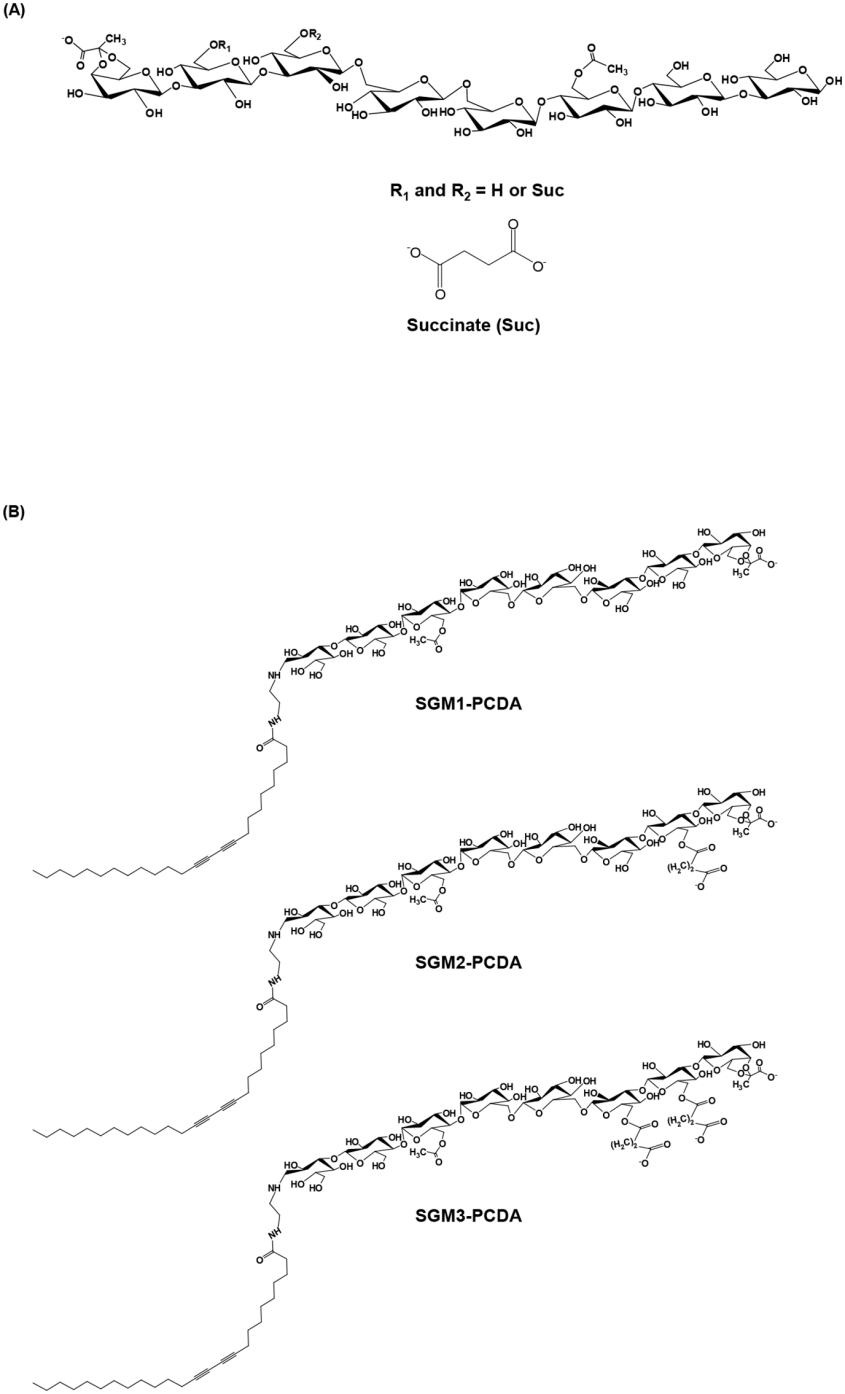
Chemical structures of succinoglycan monomers and pentacosa-10,12-diynoyl succinoglycan monomers. (A) Succinoglycan monomers with several substituents such as pyruvyl, acetyl, and succinyl groups. At the third sugar residue from the reducing end, the acetyl group is located; up two succinyl groups are located at the sixth and seventh sugar residue; and the pyruvyl group is linked to the eighth sugar residue through a 4,6-ketal linkage. (B) Pentacosa-10,12-diynoyl succinoglycan monomers pentacosadiynoylated at the reducing galactose of succinoglycan monomers.

Isolation and purification of succinoglycan monomers from *S*. *meliloti* Rm 1021 were carried out using size exclusion chromatography and anion exchange chromatography with procedure as described in Materials and Methods. The chemical structure of succinoglycan monomers (SGM) are shown in Fig 1A. The SGM were classified into three types (SGM1, SGM2, or SGM3) according to the number of succinyl groups present [7]. The structure of each SGM was confirmed using MALDI-TOF MS [29].

### Synthesis of pentacosa-10,12-diynoyl succinoglycan monomers

**Fig 2:**
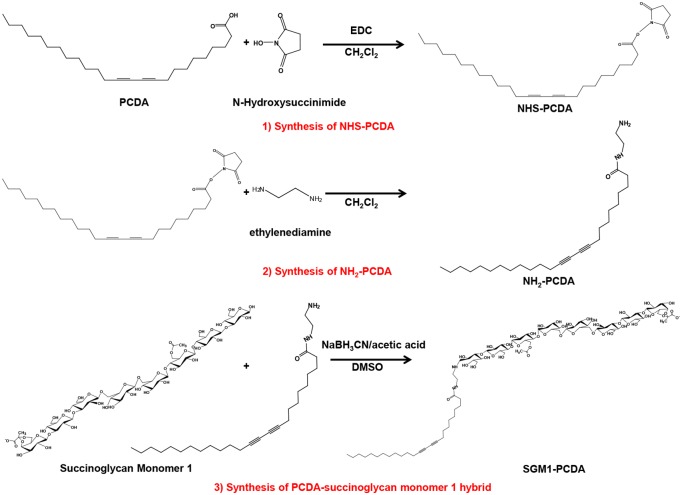
Synthesis of pentacosa-10,12-diynoyl succinoglycan monomer 1. Synthesis of pentacosa-10,12-diynoyl succinoglycan monomer 1 was achieved by three synthetic procedures. (1) *N*-Hydroxysuccinimide ester of 10,12-pentacosadiynoic acid (NHS-PCDA) was synthesized using cross-linker EDC and NHS. (2) *N*-(2-aminoethyl)-10,12-pentacosadiynamide (NH_2_-PCDA) was synthesized using NHS-PCDA and ethylenediamine. (3) Pentacosa-10,12-diynoyl succinoglycan monomer 1 (SGM1-PCDA) was synthesized through reductive amination with succinoglycan monomer 1 (SGM1) and NH_2_-PCDA using the reducing agent, sodium cyanoborohydride.

The PCDA functionalized with carbohydrate molecules, pentacosa-10,12-diynoyl succinoglycan monomers (SGM-PCDA) were synthesized from NH_2_-PCDA (*N-*(2-aminoethyl)-10,12-pentacosadiynamide) and succinoglycan monomers, as shown in Fig 2. The synthesis of NH_2_-PCDA was carried out by the reaction using NHS-PCDA and ethylenediamine and the structure was confirmed by ESI-MS. MS (ESI+): [M+H]^+^ 417.3 (calculated for C_27_H_48_N_2_O: 416.38).

**Fig 3:**
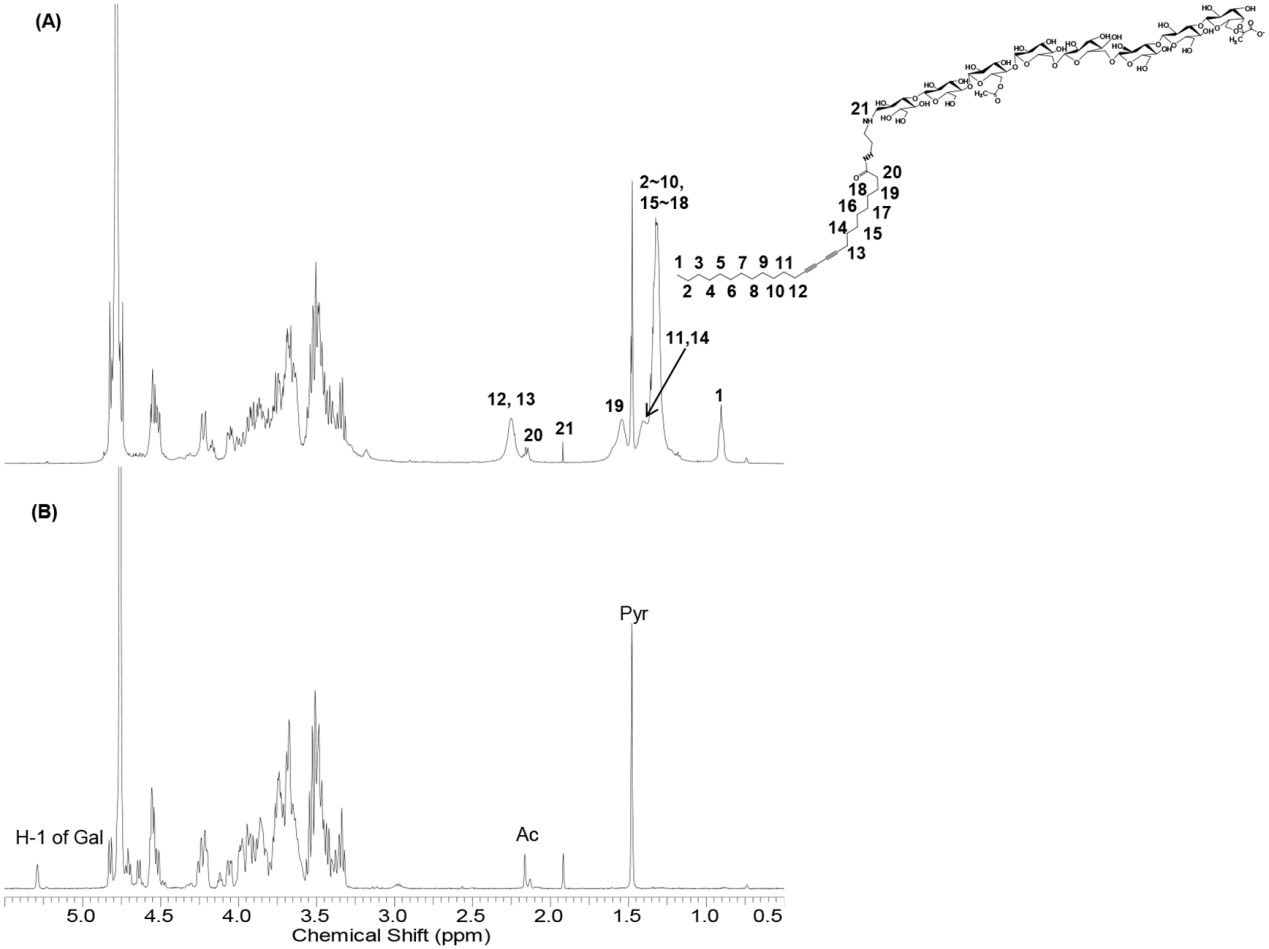
^1^H NMR spectra (D_2_O). (A) ^1^H NMR spectrum of SGM1-PCDA. After the reductive amination reaction, the characteristic peak at reducing sugar disappeared. Other peaks corresponding to protons of PCDA part and original succinoglycan backbone. (B) ^1^H NMR spectrum of original succinoglycan monomer 1.

The product, SGM-PCDA, synthesized from NH_2_-PCDA and SGM, was analyzed by MALDI-TOF mass spectrometry (S1 Fig) and NMR spectroscopy (Fig 3). The chemical structure of SGM-PCDA is shown in Fig 1B. The molecular ions ([(SGM1-PCDA)-H_2_O-2H+Na]^-^, [(SGM2-PCDA)-H_2_O-2H+Na]^-^, and [(SGM3-PCDA)-H_2_O-2H+Na]^-^) were shown at *m/z* 1828, 1928.2, and 2028 in negative-ion mode (S1 Table). This result indicates that the mass differences of SGM1-PCDA, SGM2-PCDA, and SGM3-PCDA are matched to *m/z* 100 due to *O*-ester linked succinyl residue. Furthermore, the mass patterns such as the loss of one acetyl and galactose were similar to those of original SGM.

The NMR spectroscopy was used to explicate the structure of novel PCDA functionalized with succinoglycan monomers. In the case of SGM1, the proton chemical shifts from 3.30 ppm to 5.00 ppm correspond to protons of the original succinoglycan backbone, and the H-1 peak of characteristic reducing galactose appeared at 5.28 ppm (Fig 3B). After the reductive amination reaction, the particular peak at 5.28 ppm disappeared, and other peaks corresponding to protons of PCDA part are shown (Fig 3A). Since succinoglycan monomers are linear carbohydrates, they could be pentacosadiynoylated at the reducing sugar. This result indicates that the hybrid molecule, SGM1-PCDA, was successfully synthesized as we desired. Additionally, ^1^H NMR spectra in case of SGM2-PCDA and SGM3-PCDA were shown as the same patterns (S2 Fig).

### Fluorescence study of PDA liposome containing SGM1-PCDA comparing with mono[6-deoxy-6-(pentacosa-10,12-diynyl amidomethyl)]-β-cyclodextrin

**Fig 4:**
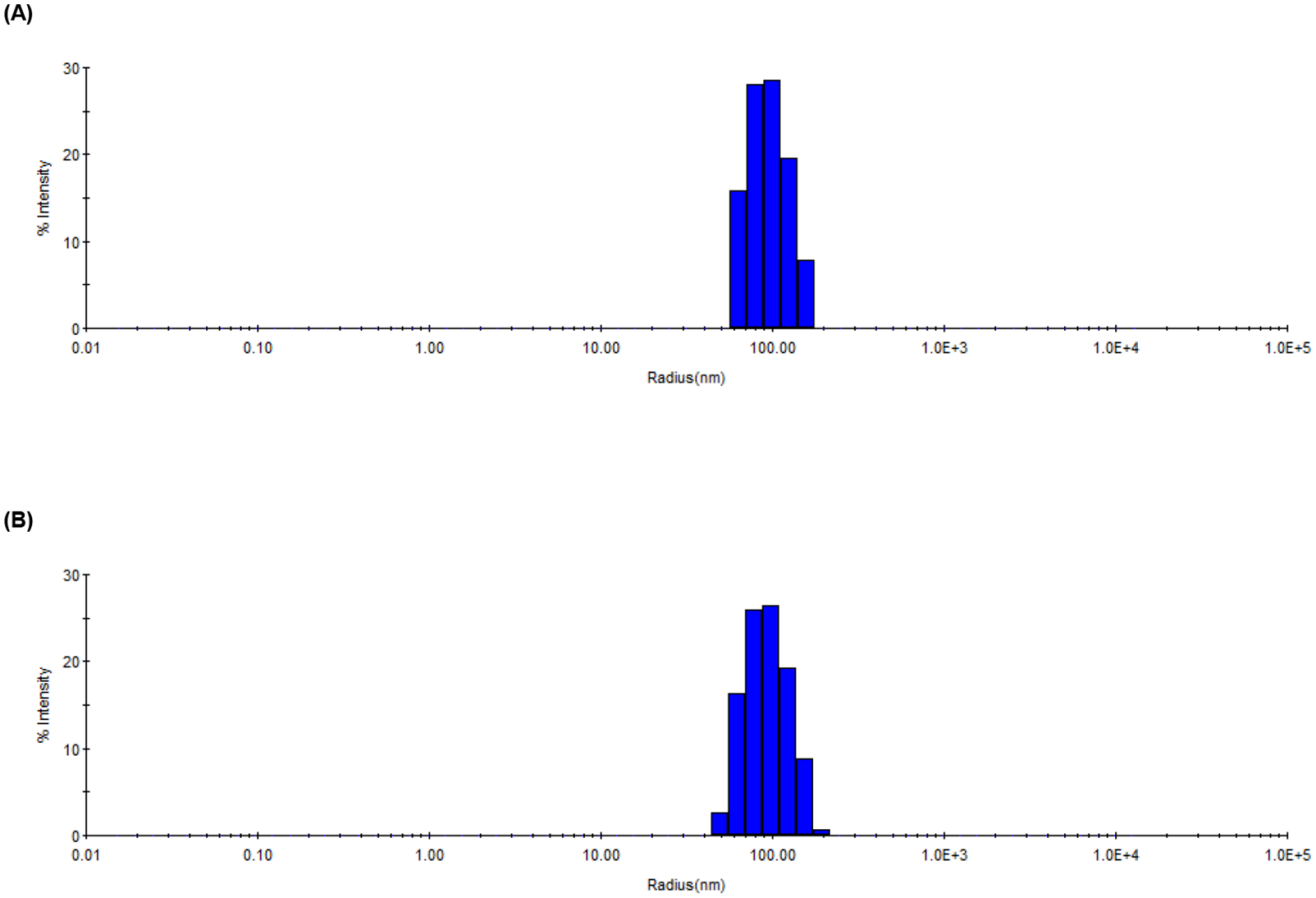
DLS profiles. Prior to polymerization of (A) Pure PDA vesicles and (B) Liposomes containing SGM1-PCDA.

**Fig 5:**
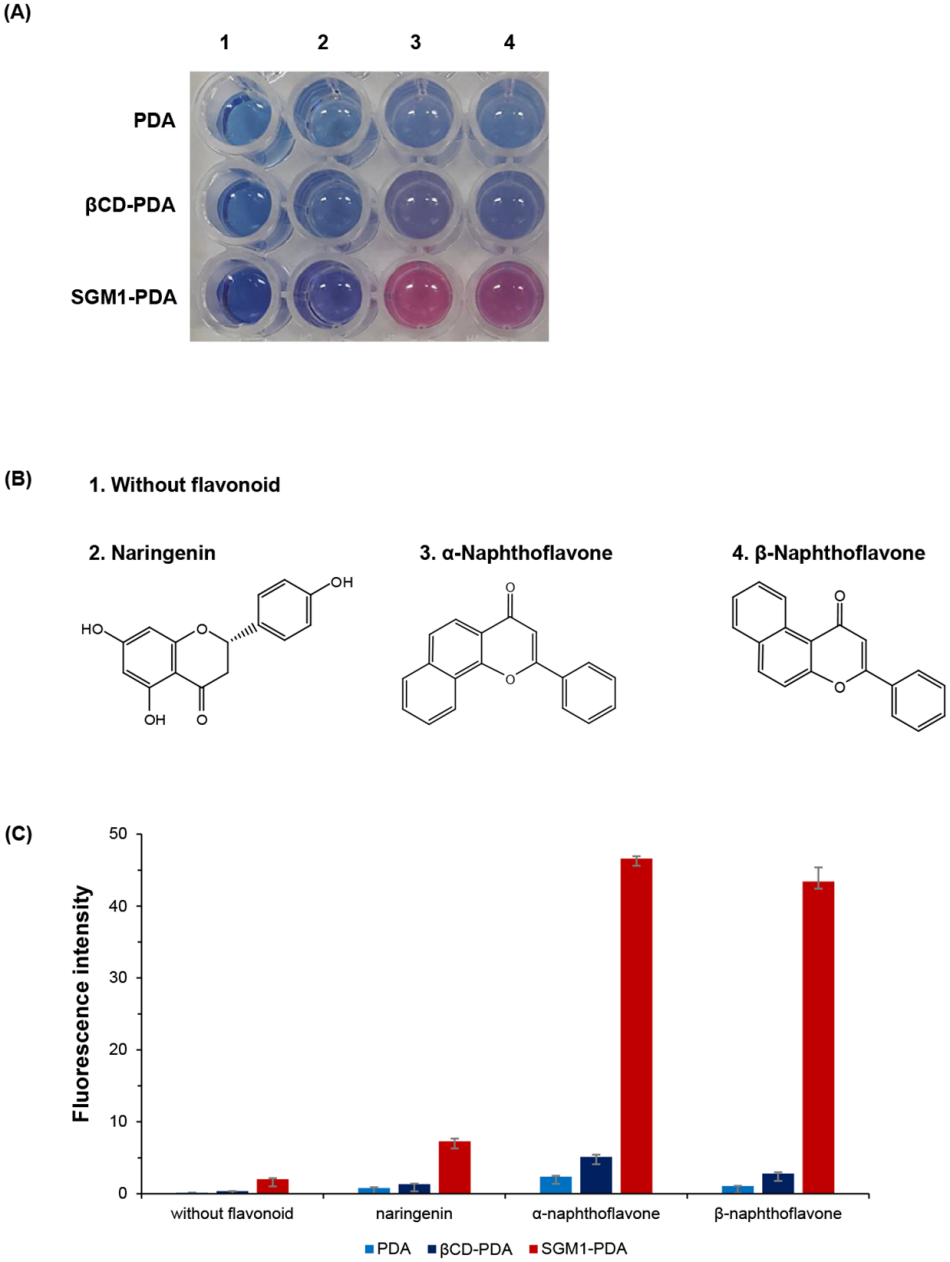
Effect of flavonoids on SGM1-PDA. (A) Pure PDA vesicles and the color change of βCD-PDA and SGM1-PDA in the presence of flavonoids; (1) without a flavonoid, (2) naringenin, (3) α-naphthoflavone, and (4) β-naphthoflavone. (B) The chemical structures of flavonoids (naringenin, ANF, and BNF). (C) The fluorescence intensities at 560nm of samples in the presence of flavonoids (2 mM). The reaction system was mixture of 20 μL flavonoids solution and 80 μL liposome solution (1 mM) of βCD-PDA or SGM1-PDA.

Before polymerization via UV irradiation, the size of the PDA liposomes was investigated using dynamic light scattering (DLS), and the size distribution is shown in Fig 4. The diameter of the SGM1-PDA is slightly increased comparing with that of pure PDA liposomes. The average diameters of the modified and original PCDA liposomes were determined as 106 and 86 nm, respectively. This result indicates that the bulky and flexible head group by SGM1 might contribute on the size increase and our desired liposome was well prepared. Since color change of blue to red occurs by π-orbital twisted on PDA array [30], the blue-phase SGM-PDA can be changed into red-phase when there is a specific interaction with target molecules. As shown in Fig 5A, when the flavonoids were added to the SGM1-PDA, the color change was definitely accompanied. Since βCD is well known to host hydrophobic molecules including flavonoids, we also carried out colorimetric detection test with βCD-PDA as other host. In the presence of α-naphtoflavone (ANF) and β-naphtoflavone (BNF), SGM1-PCDA induces the color change derived from flexible head group, whereas βCD-PCDA shows no special effect. Although βCD forms inclusion complexes with flavonoids [4, 31], the complex may not induce the π-orbital twist on PDA array derived from rigid structure of βCD [32]. In case of naringenin, because of its relative hydrophilicity compared with ANF and BNF, the color transition was not observed in both of βCD-PDA and SGM1-PDA. Moreover, we investigated the fluorescence changes in the presence of target compounds. As shown in Fig 5C, we could confirm the differential fluorescence intensity of SGM1-PDA comparing with those of pure PDA and βCD-PDA. This result indicates that colorimetric detection system using SGM1-PCDA is more effective method to detect for flavonoid than PDA liposome with a rigid βCD head group. For these reasons, we performed colorimetric test with SGM2-PDA and SGM3-PDA having linear structure as host molecules.

### Color transition on flavonoids with SGM1-PDA, SGM2-PDA and SGM3-PDA

**Fig 6:**
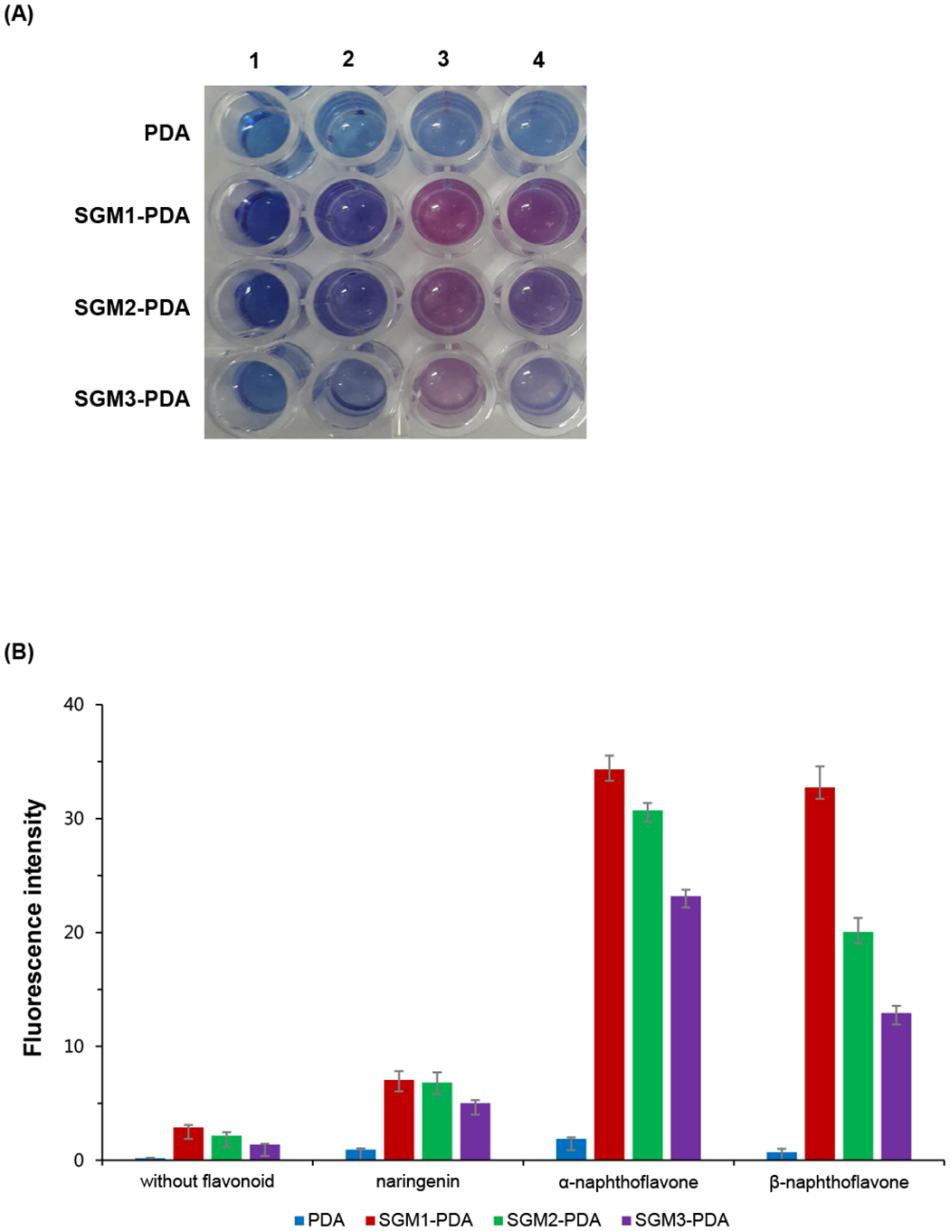
Effect of succinoglycan monomers. (A) Pure PDA vesicles and the color change of SGM1-PDA, SGM2-PDA, and SGM3-PDA. (B) The fluorescence intensities at 560 nm in detection system with 20 μL flavonoids solution (2 mM) and 80 μL polymerized liposome solution (1 mM).

As shown in Fig 6, the color change was observed with the naked eye (Fig 6A) and we also confirmed fluorescence changes at 560 nm in case of SGM2-PDA and SGM3-PDA (Fig 6B). This result supports our hypothesis; linear structure with flexibility may make more interaction with target molecules and induce steric repulsion on the PDA liposome. Moreover, the red color transition occurred more powerfully in the SGM1-PDA than SGM2-PDA and SGM3-PDA. SGM2 and SGM3 could take more potential structures to be able to form intramolecular hydrogen bonding due to the succinyl group. Based on intramolecular conformational change, it is considered that bent molecular geometry of SGM2 and SGM3 may have a loss of interaction opportunity. In the previous study, SGM1 has also shown the highest efficiency for the solubility enhancement of a hydrophobic drug, pindolol among SGM. [9]. In our case, the interaction response with flavonoids appeared more significantly in the SGM1-PDA.

### Visible spectroscopic and fluorescence monitoring of color transition on β-naphthoflavone

**Fig 7:**
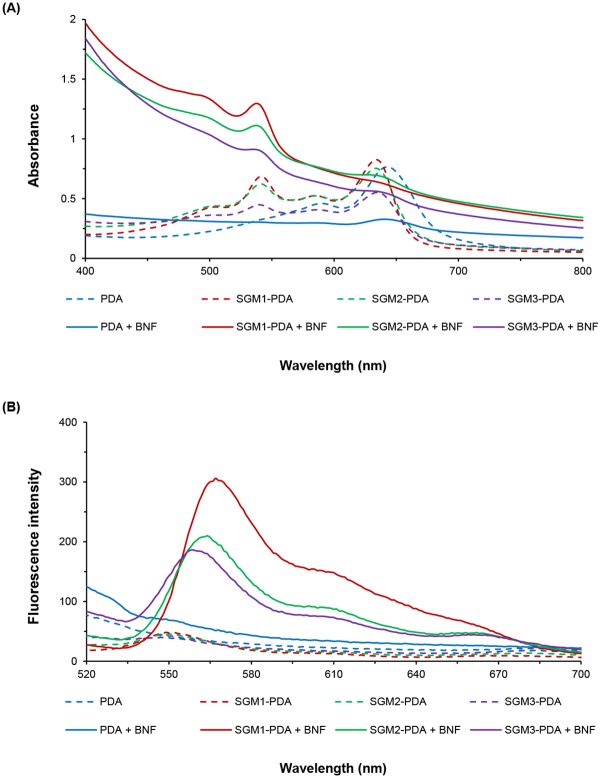
Visible spectroscopic and fluorescence changes on β-naphthoflavone. (A) UV-Vis spectra of SGM-PDA. Each solid line and dotted line represents a condition with BNF (0.4 mM) and without BNF. (B) Emission change of SGM-PDA. The study was performed before (dotted line) and after (solid line) addition of 2 mM BNF.

To confirm red-phase transition of the PDAs liposome, visible spectroscopic and fluorescence changes were further monitored when a highly hydrophobic flavonoid, β-naphthoflavone was added to the SGM-PDA. Upon addition of BNF, a decrease of absorbance was observed at 640 nm, while an increase of absorbance appeared at 550 nm (Fig 7A). As shown in Fig 7B, the fluorescence spectra of SGM-PDA appeared an increased fluorescence intensity with BNF at 560 nm. By monitoring visible spectroscopic response and emission change, we can estimate once again that SGM1-PDA shows more expressive response on flavonoids interaction. This result indicates that our detection system demonstrates an archetypal color transition as sensor using PCDA.

### Differential fluorescence intensity on the concentration of β-naphthoflavone and CR% change in time

**Fig 8:**
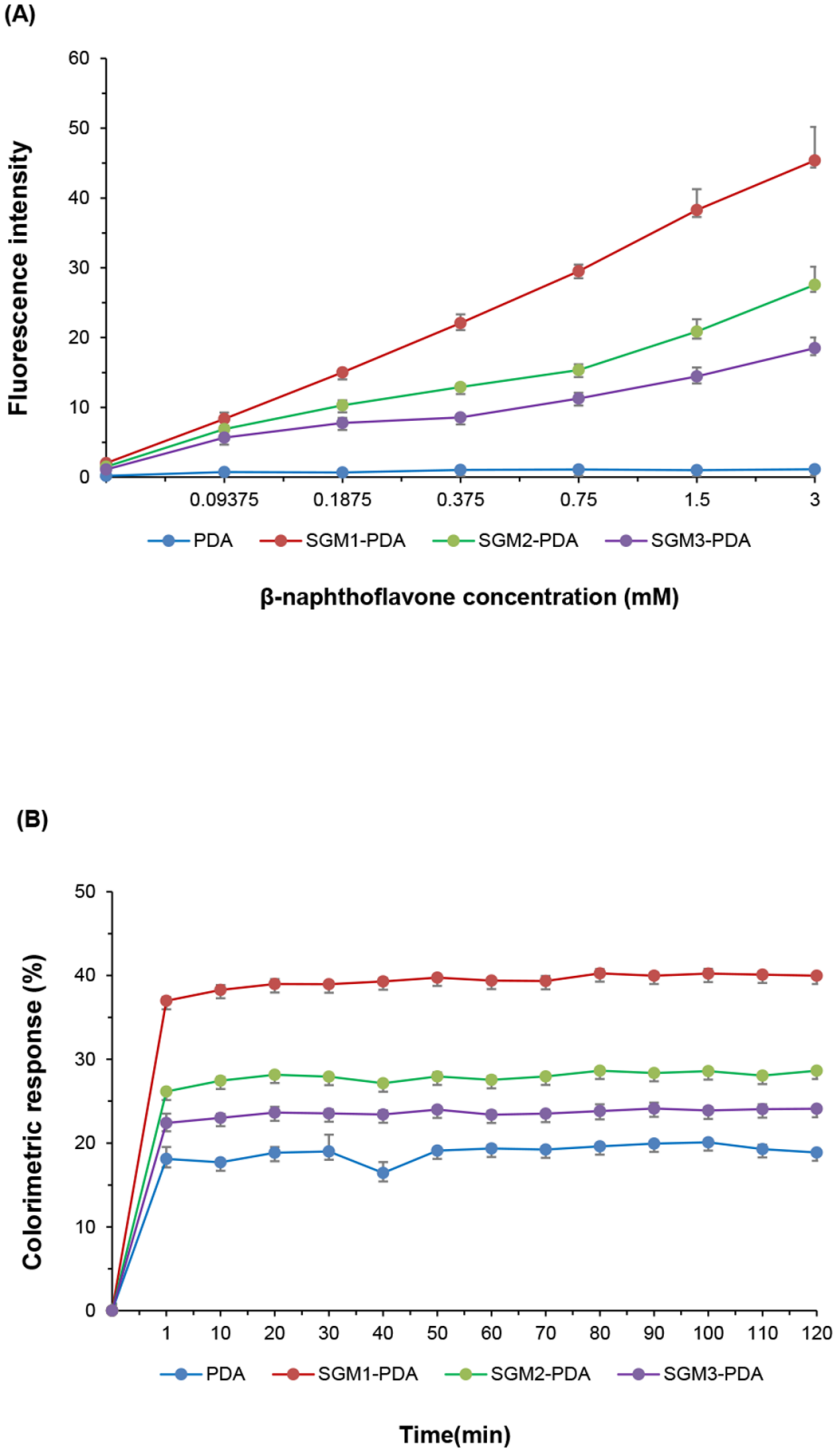
Fluorescence intensity changes from varying concentrations of β-naphthoflavone and CR% change in time. (A) Changes of fluorescence intensity on SGM-PDA with various concentrations of BNF (0.09375, 0.1875, 0.375, 0.75, 1.5 and 3 mM). (B) Kinetic study for the CR% change of SGM-PDA with 2 mM concentration of BNF.

To confirm sensitivity of colorimetric detection system using SGM-PCDA, we performed fluorescence study on BNF concentration. As shown in Fig 8A, the original PDA liposome did not show any fluorescence. On the other hand, PDA liposome modified with succinoglycan monomers showed the increased fluorescence intensity as a function of BNF concentration. The SGM1-PDA showed the largest fluorescent enhancement with BNF and the emission change of up to 40-fold was observed on addition of BNF (3 mM). In addition, the detection limit with naked eye was observed at 187.5 μM of BNF concentration. Throughout measurement of fluorescence change, we could confirm color transition at lower BNF concentration, as compared with that observed with the naked eye. This means that our colorimetric detection system is capable of detecting flavonoids in micro molar concentrations.

We also investigated kinetics for CR% change of SGM-PDA with BNF (2 mM). As shown in Fig 8B, we obtained the curve of CR% vs. time when BNF was added to pure PDA liposomes and SGM-PDA. Although the CR% of pure PDA was increased upon addition of BNF, the CR% less than 20% showed no color change. Whereas, the color changes of SGM1-PDA, SGM2-PDA, and SGM3-PDA were clearly observed, and the CR% values are determined as 36.9, 26.1, and 22.4, respectively. Furthermore, the colorimetric response appeared within 1 min, and the response was maintained for 2 h. This result indicates that the present system could be rapid and robust detection methods for BNF.

### Effect of hydrophobicity on flavonoid capturing

**Fig 9:**
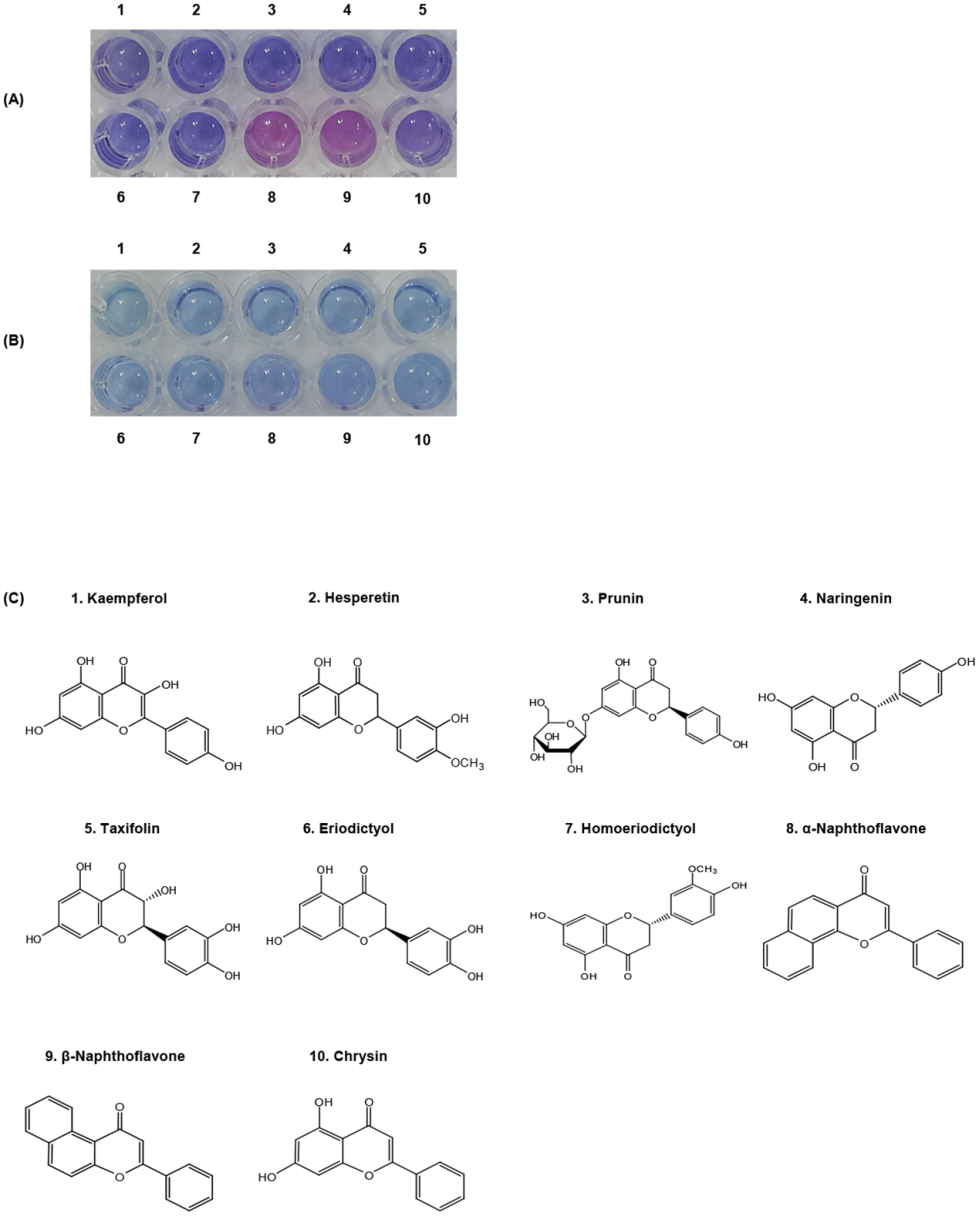
Effect of hydrophobic interactions. Photographs of (A) SGM1-PDA and (B) Pure PDA liposomes in the presence of various flavonoids (1mM). (C) The structure of each flavonoid such as kaempferol, hesperetin, prunin, naringenin, taxifolin, eriodictyol, homoeriodictyol, ANF, BNF, and chrysin.

As shown in Figs 5 and 6, the color transition was not observed in the presence of naringenin. On the other hand, the red-phase transition appeared in both of adding highly hydrophobic flavonoids such as ANF and BNF. Structurally, naringenin has more hydrophilicity relative to ANF and BNF due to three hydroxyl groups. Therefore, we investigated the effect of hydrophobicity on flavonoid capturing with ten different flavonoids. The SGM1-PDA showed a selective blue to red transition in the presence of ANF and BNF (Fig 9A). On the other hand, the pure PDA liposome did not show any color transition after addition of flavonoids (Fig 9B). The structure of each flavonoid including kaempferol, hesperetin, prunin, naringenin, taxifolin, eriodictyol, homoeriodictyol, ANF, BNF, and chrysin was displayed in Fig 9C. The respective log *P* values using ACD/Percepta Platform are also listed in Table 1, which are used to measure the hydrophobicity of a compound [33]. We can confirm that ANF and BNF have much higher log *P* values (4.79) than other flavonoids with some hydroxyl groups. This result indicates that SGM1-PCDA could induce the color change with highly hydrophobic flavonoids through hydrophobic interactions.

**Table 1:** Predicted log *P* values of flavonoids.

Flavonoid	log *P*	Molecular wt
**Kaempferol**	**2.05**	**286.24**
**Hesperetin**	**2.90**	**302.28**
**Prunin**	**0.82**	**434.4**
**Naringenin**	**3.19**	**272.26**
**Taxifolin**	**1.82**	**304.3**
**Eriodictyol**	**2.59**	**288.27**
**Homoeriodictyol**	**2.90**	**302.39**
**α-Naphthoflavone**	**4.79**	**272.3**
**β-Naphthoflavone**	**4.79**	**272.3**
**Chrysin**	**2.88**	**254.2**

## Discussion

### Linear oligosaccharides unit produced from *S*. *meliloti*

Cell-associated carbohydrates of Rhizobiaceae family are known to be involved in bacterium-plant interactions occurring both in pathogenesis and symbiosis [34]. Those microbial carbohydrates include extracellular polysaccharides (EPS), lipopolysaccharides, and periplasmic glucans. EPS provide a survival benefit to microbes by protecting them from dehydration, and are buffers against changes in surrounding environment. EPS also prevent invasion by other pathogenic bacteria [35]. EPS have applications in cosmetics, pharmaceutics, food, and paper protective wrappings [36]. Succinoglycans from the family of EPS have an octasaccharide subunit substituted with a succinyl, acetyl, and pyruvyl group. Succinoglycans have had uses in enhancement of solubility on poorly soluble drugs through complexes and as chiral additives [8–11]. In addition, flavonoids have been reported that they could provide the expression of bacterial *nod* genes, which promote root nodulation in the nitrogen fixation soil bacteria [37]. Based on the physico-chemical properties of succinoglycans, our study for the flavonoid detection system was designed.

### Accomplishment of flavonoid detection using PCDA derivatives

PDA liposomes have the property of blue to red color change occurring by the delocalizing π-orbital of polymerized backbone from an external physical stress such as heat, pH, temperature, and intermolecular binding. Since blue PDAs are non-fluorescent and red PDAs are strongly fluorescent at a specific wavelength, polydiacetylene-based chemosensors using PCDA can be monitored by visible spectroscopy or naked eye making them unique detectors. Using this property, sialic acid attached PCDA was designed and used for the colorimetric detection of influenza virus. The main idea was that the hemagglutinin (HA) and neuraminidase (NA) on the virus surface could recognize and bind to terminal glycosides of sialic acid [38, 39].

In our study, the investigation of colorimetric detection for flavonoids was performed using synthesized pentacosa-10,12-diynoyl succinoglycan monomers. In addition, we also confirmed the structural benefit of succinoglycan monomers from *S*. *meliloti* by comparing liposomes containing SGM1-PCDA and βCD-PCDA. As shown in Fig 5, pure PDA liposomes and βCD-PDA liposomes did not show any changes in fluorescence intensity, however SGM1-PDA did in presence of flavonoids. The resultant color changes were easily observed with a naked eye.

The selectivity of PDA liposome modified with succinoglycan monomers could be considered throughout the investigation of ten different flavonoids (Fig 9A). Considering that ANF and BNF have the highest log *P* values among them, the high hydrophobicity in flavonoid structures could make the hydrophobic interaction with SGM1-PDA stable (Table 1). The molecular interaction contributes as a factor to distort the π-orbital on PDA array and results in color transition. Our study indicates that the colorimetric detection system with SGM-PCDA is suitable to detect highly hydrophobic flavonoids, α-naphthoflavone and β-naphthoflavone.

**Fig 10:**
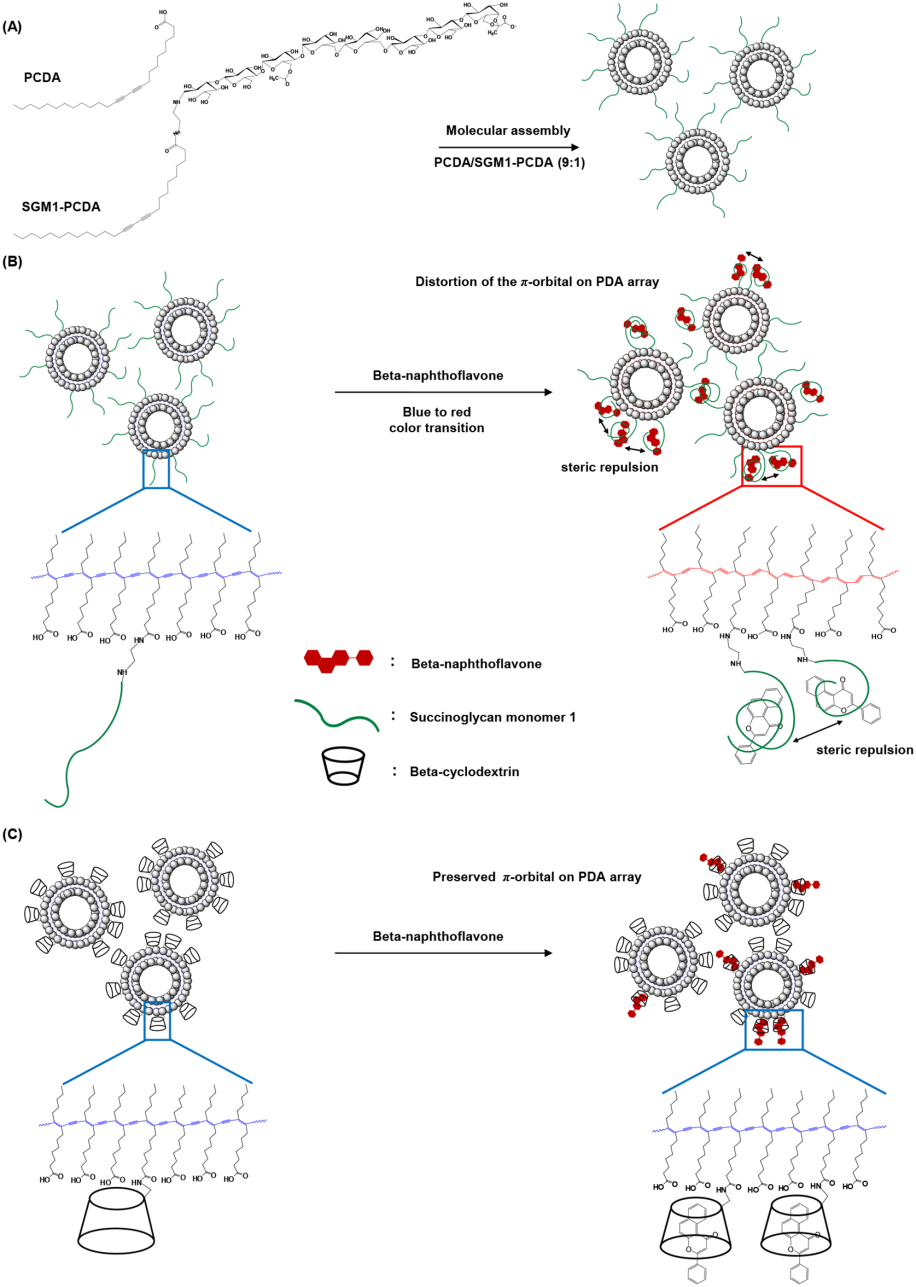
Hypothetical scheme on flavonoid capturing. (A) A schematic representation of self-assembly with SGM1-PCDA and PCDA. (B) Color transition by steric effects between adjacent SGM1-BNF complexes. (C) Preserved π-orbital on PDA array of βCD-PDA.

Based on our results, we have introduced a theoretical model for details of flavonoid capturing in our system. Schematic illustration of a self-assembly by PCDA and SGM1-PCDA were shown as Fig 10A and 10B. Recent study has reported that the intensity of PDA color transition could be due to a steric repulsion rather than the strength of the binding force [40]. The conformational change of PDA liposome through steric repulsion induced by intermolecular complexes produces the blue to red color transition [41, 42]. In the present study, SGM1 has a recognition to flavonoids through an induced fit adjustment of linear glucans [9], and the recognition event will change the form factor of SGM1-flavonoid complex. Since the steric repulsion between adjacent SGM1-flavonoid complexes will produce good enough stress to induce a π-orbital twist on PDA array, SGM1-PDA produces the sensory signal generation (Fig 10B). However, in the case of βCD-PCDA, the target flavonoid is captured into the cavity of rigid βCD and there is no steric change before and after the molecular complex event. The complex could not affect the π-orbital twist on PDA array and thereby no sensory signal can be generated (Fig 10C). Therefore, we suggest that the flexible SGM1 can act as a transmissible antenna to induce the steric repulsion on the PDA liposome. Likewise, SGM2-PCDA and SGM3-PCDA play the role for flavonoids (Fig 6). Summarizing our observations, the PDA-based colorimetric detection using SGM-PCDA can be an effective yet user-friendly detection method for some highly hydrophobic flavonoids.

## Conclusion

We isolated and purified the linear oligosaccharides, succinoglycan monomers, from *S*. *meliloti* Rm 1021. With PCDA, we synthesized SGM-PCDA which was then characterized using NMR spectroscopy and MALDI-TOF mass spectrometry. Through fluorescence and colorimetric studies using the artificial PDAs derivative with SGM-PCDA, we verified the functionality as a colorimetric detection for some highly hydrophobic flavonoids such as ANF and BNF. Moreover, it is suggested that the flexible host can be more effective in steric repulsion for the triggering of PDA array than rigid host. The present detection strategy for flavonoids has potential in terms of easy, time-saving and visible method differently from the established one. By extension, this system will be applied to detecting other hydrophobic compounds besides flavonoids.

## Supporting Information

S1 Fig MALDI-TOF mass spectra of SGM-PCDA (negative mode, THAP matrix). (TIF)

S2 Fig ^1^H NMR spectra. Pentacosa-10,12-diynoyl succinoglycan monomer 2 (top). Pentacosa-10,12-diynoyl succinoglycan monomer 3 (bottom).
(TIF)

S1 Table Measured and calculated mass units of the m/z peaks of SGM-PCDA. (DOCX)
