# Supplementary figures and images for: Colorimetric Detection of Some Highly Hydrophobic Flavonoids Using Polydiacetylene Liposomes Containing Pentacosa-10,12-diynoyl Succinoglycan Monomers
Source: PLoS One. 2015 Nov 23;10(11):e0143454. doi: 10.1371/journal.pone.0143454 (PMC4658141; doi:10.1371/journal.pone.0143454)

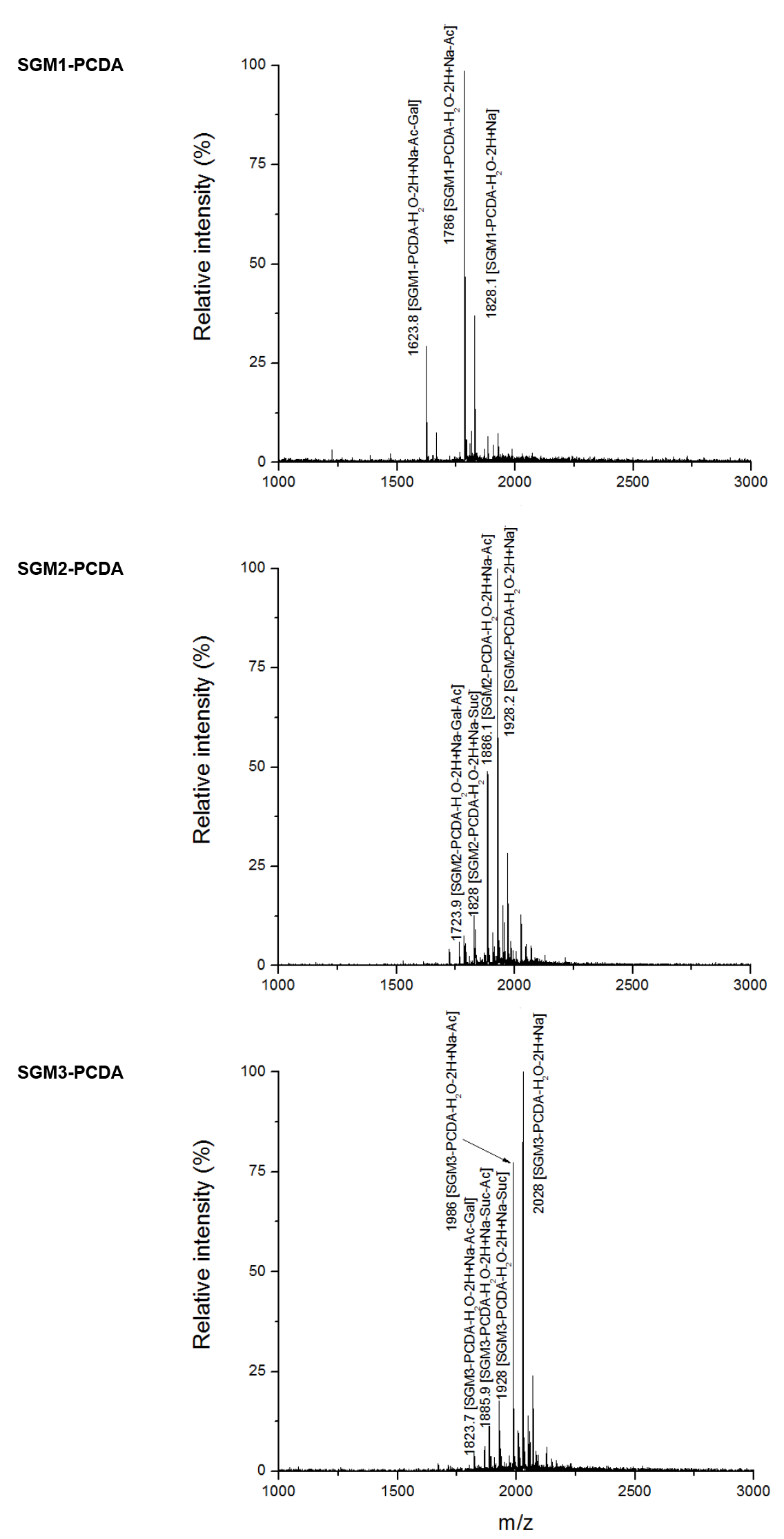

Supplement: S1 Fig — (TIF) [file pone.0143454.s001.TIF]

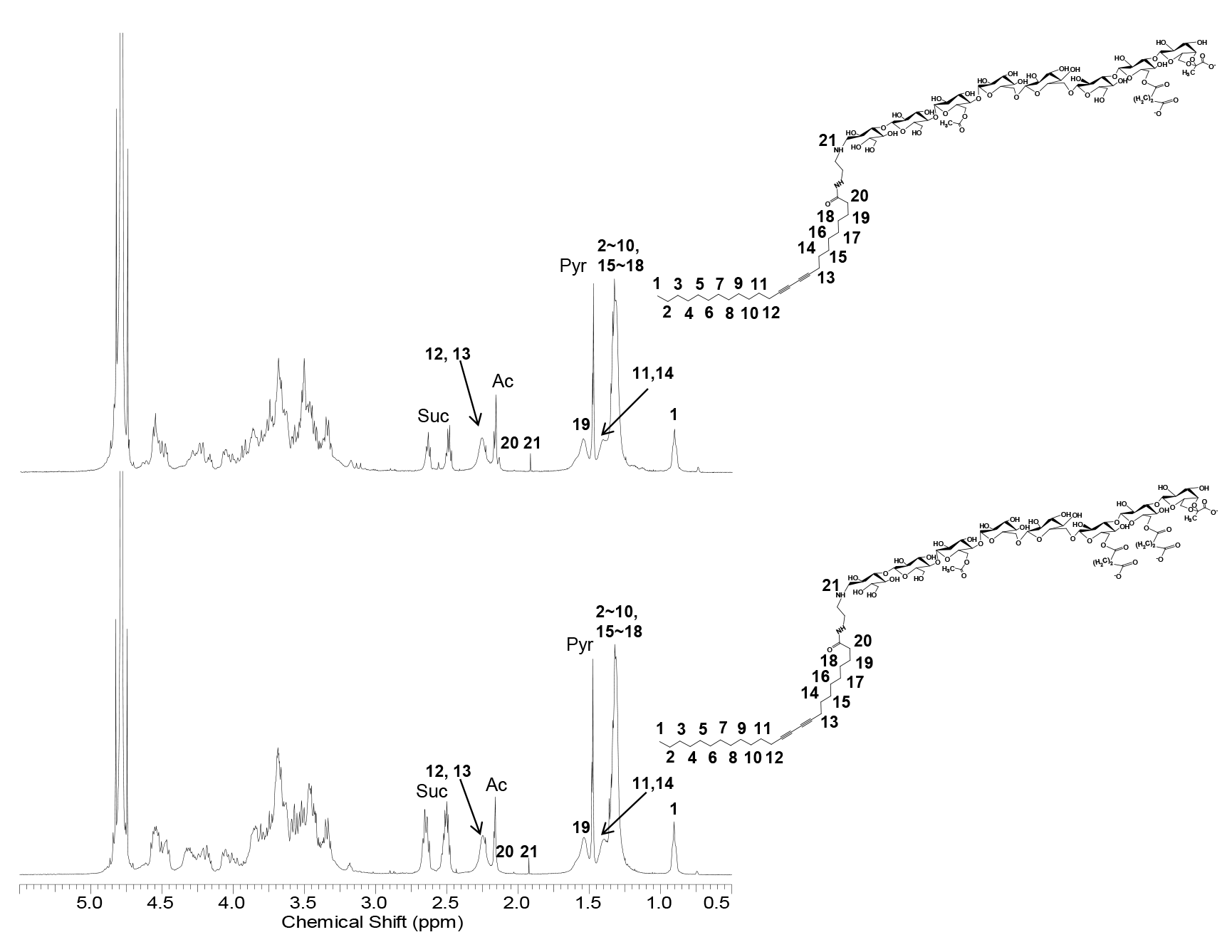

Supplement: S2 Fig — Pentacosa-10,12-diynoyl succinoglycan monomer 2 (top). Pentacosa-10,12-diynoyl succinoglycan monomer 3 (bottom). (TIF) [file pone.0143454.s002.TIF]
