# Supplementary material for: Colorimetric Detection of Some Highly Hydrophobic Flavonoids Using Polydiacetylene Liposomes Containing Pentacosa-10,12-diynoyl Succinoglycan Monomers
Source: PLoS One. 2015 Nov 23;10(11):e0143454. doi: 10.1371/journal.pone.0143454 (PMC4658141; doi:10.1371/journal.pone.0143454)
Supplement: S1 Table — (DOCX) [file pone.0143454.s003.docx]

|  | **Calculated m/z** | **Measured m/z** | **Fragment ion assignment** |
| --- | --- | --- | --- |
| **SGM1-PCDA** | 1621.7 | 1623.8 | [M-H_2_O-2H+Na-Ac-Gal] ^-^ |
|  | 1783.8 | 1786 | [M-H_2_O-2H+Na-Ac] ^-^ |
|  | 1825.8 | 1828.1 | [M-H_2_O-2H+Na] ^-^ |
| **SGM2-PCDA** | 1721.8 | 1723.9 | [M-H_2_O-2H+Na-Ac-Gal] ^-^ |
|  | 1825.9 | 1828 | [M-H_2_O-2H+Na-Suc] ^-^ |
|  | 1883.9 | 1886.1 | [M-H_2_O-2H+Na-Ac] ^-^ |
|  | 1925.9 | 1928.2 | [M-H_2_O-2H+Na] ^-^ |
| **SGM3-PCDA** | 1821.2 | 1823.7 | [M-H_2_O-2H+Na-Ac-Gal] ^-^ |
|  | 1883.3 | 1885.9 | [M-H_2_O-2H+Na-Ac-Suc] ^-^ |
|  | 1925.3 | 1928 | [M-H_2_O-2H+Na-Suc] ^-^ |
|  | 1983.3 | 1986 | [M-H_2_O-2H+Na-Ac] |
|  | 2025.3 | 2028 | [M-H_2_O-2H+Na] ^-^ |
